# Maternal Sociodemographic Parameters: Impact on Trace Element Status and Pregnancy Outcomes in Nigerian Women

**DOI:** 10.3329/jhpn.v29i2.7858

**Published:** 2011-04

**Authors:** Emmanuel I. Ugwuja, Emmanuel I. Akubugwo, Udu A. Ibiam, Onyechi Obidoa

**Affiliations:** ^1^Department of Chemical Pathology, Faculty of Clinical Medicine, Ebonyi State University, PMB 053 Abakaliki, Nigeria; ^2^Department of Biochemistry, Abia State University, Uturu, Nigeria; ^3^Department of Biochemistry, Faculty of Biological Sciences, Ebonyi State University, PMB 053 Abakaliki, Nigeria; ^4^Department of Medical Biochemistry, University of Nigeria, Nsukka, Nigeria

**Keywords:** Maternal nutrition, Morbidity, Pregnancy outcomes, Socioeconomic status, Trace elements, Nigeria

## Abstract

To determine the impact of socioeconomic status on plasma trace element status and pregnancy outcomes, 349 pregnant women, aged 15-40 years (mean 27.04 ±2.75 years), recruited at ≤25 weeks (mean 21.76±3.12 weeks) gestational age, were followed up till delivery during which maternal and foetal outcomes were recorded. Plasma copper, iron, and zinc were determined using atomic absorption spectrophotometer while maternal sociodemographic data were obtained using a questionnaire. Except for copper, lower plasma iron and zinc were significantly (p<0.05) higher in women from socioeconomically-disadvantaged groups. Both adverse maternal health and foetal outcomes also seemed to be more prevalent in socioeconomically-disadvantaged women, although without a definite trend. This study has shown that, in economically-disadvantaged setting of developing countries, maternal socioeconomic status impacts on maternal trace element (copper, iron, and zinc) status and health and foetal outcomes.

## INTRODUCTION

Maternal socioeconomic status and non-modifiable, non-biological factors that affect mental and physical well-being (1) have been associated with maternal nutrition and pregnancy outcomes (2, 3). Although it is increasingly acknowledged that societal factors play a significant role in micronutrient status and pregnancy outcomes (1, 3), studies on impacts of socioeconomic status on pregnancy outcomes have produced conflicting results (4, 5). For instance, the risk of preterm birth has been reported in mothers of low socioeconomic status (6). In Danish and Norwegian populations, the risk of preterm birth was reported to have an inverse association with educational level of mothers (7). Morgen *et al.* did not find any association between risk of preterm birth and other indicators of socioeconomic status, such as household income and parental occupation (2). A study in Germany found that women with the lowest level of education had a significantly-elevated risk of small-for-gestational age newborns compared to women with the highest level of education (8), with the distribution of factors known to influence intrauterine growth varying with educational level. There has also been controversy over the influence of unemployment in the family on pregnancy outcomes. While some studies have shown associations of unemployment with preterm delivery (9, 10), low birthweight (LBW) (11), small-for-gestational age (12), and a higher perinatal mortality rate (11), others have shown opposite results (13, 14). Additionally, deficiencies of trace elements, including copper and zinc, have been associated with maternal morbidity, such as hypertension (copper), infections, and diabetes mellitus (zinc) without significant effects on foetal outcomes (15). Adverse foetal outcomes have been recognized to constitute an important public-health problem because several chronic diseases of public-health significance have been traced to adverse intrauterine and early life (16, 17). Although a high prevalence of deficiencies of trace elements (copper, iron, and zinc) has been reported among Nigerian pregnant women (18), there is a paucity of data on the role of maternal socioeconomic status on plasma levels of these trace elements and pregnancy outcomes. The present study was, therefore, conducted to determine the impact of maternal sociodemographic parameters on plasma iron, copper and zinc level and pregnancy outcomes in Nigerian pregnant women.

## MATERIALS AND METHODS

### Study settings and sample

The study was conducted among pregnant women attending the antenatal clinic of the Department of Obstetrics and Gynaecology of the Federal Medical Centre, Abakaliki, one of the referral tertiary health institutions in southeastern Nigeria, with an average delivery of 400 per annum. Abakaliki and the environs are inhabited mainly by subsistence-level population. Their main occupation is subsistence-level farming—mainly yam and cassava—with some animal husbandry. Other professions and/or activities, such as civil service, trading, artisanry, and stone quarrying, are also practised. The transmission of malaria is intense and occurs throughout the year (perennial). Three hundred and fifty-one consecutive women, aged 15-40 years (gestational age ≤25 weeks), who gave their consent to participate in the study, were recruited during July 2007–September 2008. Women with chronic disease, women who were HIV-seropositive, and women with multiple pregnancies were excluded from the study.

Sets of structured questionnaire were used for collecting sociodemographic data of the participants. Their weights and heights (metre) were measured in light clothing without shoes and standing erect against a pre-marked scale attached to the weighing balance, and body mass index (BMI) (kg/m^2^) was calculated. Five mL of fasting venous blood collected at recruitment, during 08.00-10.00 hours, were dispensed into trace element-free heparinized plastic bottles (3.0 mL) and EDTA bottles (2.0.mL) for biochemical and haematological analyses. The heparinized blood samples were centrifuged at 2,000 g for five minutes for the isolation of plasma. The plasma samples were frozen until these were analyzed. The participants were regularly followed up, based on appointment with their consultants till delivery. At every follow-up, the attending obstetricians evaluated them for anaemia (Hb <11.0 g/dL) (19), hypertension (blood pressure <140/90 mmHg), diabetes (fasting plasma glucose >7.8 mmol/L), infection due to *Helicobacter pylori* (seropositive to *H. pylori* antibody), concomitant illness, such as malaria (positive thin or thick film), upper respiratory tract infection (presence of cough/or catarrh), and urinary tract infection (UTI; positive urine protein, nitrite, and leucocytes). At delivery, baby's birth outcomes, such as weight, length, and head-circumference and for stillbirth, mode of delivery, and gestation age at delivery, were recorded. Birthweight was determined using an electronic weighing balance and recorded to the nearest 0.05 kg with the scale checked periodically throughout the study for accuracy while birth-length and head-circumference were determined by a measuring tape to the nearest 0.1 cm. A baby was considered underweight if the birthweight was ≤2.5 kg (20) and preterm if delivered at ≤37 weeks. Plasma copper, iron, and zinc were determined using atomic absorption spectrophotometric method while maternal haemoglobin concentration was estimated using cyanmethaemoglobin method (21). Plasma levels of <5.0 µmol/L (zinc), 8.0 µmol/L(copper), and 10.0 µmol/L (iron) were considered low (22).

### Analysis of data

All data, including sociodemographic and obstetrics data, were analyzed using the SPSS software for Windows (version 7.5). The differences between groups were compared using one-way analysis of variance (ANOVA). Data were expressed either as mean and standard deviation or proportion/percentage. The statistical significance was set at the p value of ≤0.05.

### Ethical approval

The Ethics and Research Committee of the Federal Medical Centre, Abakaliki, Nigeria, approved the protocol for this study.

## RESULTS

Table 1 shows the general characteristics of the pregnant women recruited at **≤**25 weeks gestation and those of the neonates at delivery. Although 351 pregnant women were recruited, one (0.3%) died early into the study, remaining 350 (99.7%). Data of women were available but samples were obtained from 349 participants as one participant declined to participate. At delivery, data of 319 (91.4%) women and their neonates were available. Data were either incomplete or were not available for the remaining 30 (8.6%) women.

**Table 1.** General characteristics of pregnant women at ≤25 weeks gestation and their neonates at delivery

Parameter	No.	Mean	SD	Range
Age (years)	350	27.04	4.75	15-40
BMI (kg/m^2^)	350	27.3	4.3	17.8-42.6
Parity	350	1.41	1.46	0-4
Gestational age (weeks)	350	21.76	3.12	11-25
Copper (µmol/L)	349	9.59	9.42	0.89-45.36
Iron (µmol/L)	349	10.25	7.69	1.79-45.12
Zinc (µmol/L)	349	9.19	9.16	0.7-67.32
Haemoglobin (g/dL)	349	10.21	1.26	6.5-13.3
Antenatal attendance	343	7.01	2.52	1-14
Duration of pregnancy (weeks)	319	39.14	1.73	33-43
Birthweight (cm)	319	3.06	0.50	2.00-4.50
Birth-length (cm)	319	50.93	4.66	41.0-80.0
Head-circumference (cm)	319	33.66	2.68	26.0-48.0

BMI=Body mass index; SD=Standard deviation

Although, in general, the women were not deficient in any of the three trace elements evaluated (mean 9.59±9.42, 10.25±7.69, and 9.19±9.16 µmol/L for copper, iron, and zinc respectively), the ranges of the trace elements varied from very low levels to very high concentrations, with copper, iron, and zinc concentrations from 0.89, 1.79, and 0.70 µmol/L to values as high as 45.36, 45.12, and 67.32 mmol/L respectively. The participants were generally anaemic with the mean haemoglobin concentration of 10.21±1.26, which is lower than the 11.0 g/dL cut-off point recommended by the World Health Organization.

Table 2 shows the impact of maternal parity and indices of maternal socioeconomic status (living accommodation, educational level, and occupation) on plasma copper, iron and zinc concentrations. Although there was no definite trend of the impact of parity and other indices of socioeconomic status on the plasma levels of copper, iron, and zinc among the pregnant women in this population, higher levels of these trace elements were found in economically-advantaged groups (e.g. women whose living accommodation were flats, women with secondary/tertiary education, and women who were civil servants/artisans), with lower plasma level found in multiparous women. Except for copper, the prevalence of lower plasma iron and zinc decreased with increased maternal education; these were not significant (p>0.05). Only 25% of the women without formal education had low plasma iron and zinc (mean 6.89±0.79 and 1.89±1.49 µmol/L respectively).

**Table 2.** Prevalence of low plasma copper, iron, and zinc in relation to sociodemographic/obstetric data

Maternal parameter	No.	Copper	Mean±SD	Iron	Mean±SD	Zinc	Mean±SD
Parity							
0	140	82 (58.6)	3.14±1.71	88 (62.8)	5.99±1.92	68 (48.6)	2.71±1.21
1	66	36 (54.5)	3.48±2.02	47 (71.2)	5.80±2.04	27 (40.9)	2.84±1.18
2	53	35 (66.0)	3.44±1.88	35 (66.0)	5.60±1.76	19 (35.8)	2.78±1.14
3	40	23 (57.5)	2.96±1.73	23 (57.5)	6.31±1.78	24 (60.0)	2.36±1.13
>3	50	27 (54.0)	3.29±1.78	29 (58.0)	5.80±1.59	22 (44.0)	2.45±1.06
Total	349	203 (58.2)	3.25±1.80	222 (63.6)	5.90±1.85	160 (45.8)	2.65±1.16
Living accommodation							
Single room	189	115 (60.8)	3.30±1.90	123 (65.1)	5.95±1.90	82 (43.4)	2.63±1.18
Flat	135	72 (53.3)	3.33±1.72	82 (60.7)	5.89±1.87	64 (47.4)	2.71±1.19
Bungalow	24	15 (62.5)	2.40±1.80	16 (66.7)	5.64±1.45	13 (54.2)	2.57±0.97
Total	348	202 (58.1)	3.24±1.80	221 (63.5)	5.90±1.85	159 (45.7)	2.65±1.16
Educational level							
None	8	5 (62.5)	2.03±0.74	2 (25)	6.89±0.79	2 (25)	1.89±1.49
Primary	42	24 (57.1)	2.79±1.65	24 (57.1)	6.22±1.92	17 (40.5)	2.59±1.13
Secondary	171	97 (56.7)	3.27±1.81	111 (64.9)	6.02±1.86	82 (48.0)	2.62±1.18
Tertiary	120	71 (59.2)	3.60±1.87	80 (66.7)	5.59±1.82	55 (45.8)	2.74±1.19
Total	341	197 (57.8)	3.30±1.81	217 (63.6)	5.89±1.86	156 (45.8)	2.65±1.17
Occupation							
Housewife	53	31 (58.5)	3.45±1.90	32 (60.4)	6.01±1.85	28 (52.8)	2.77±1.18
Student	61	40 (65.6)	3.16±1.89	40 (65.6)	5.99±1.80	29 (47.5)	3.0 ± 1.18
Civil servant	143	64 (44.8)	3.27±1.76	92 (64.3)	5.77±1.83	65 (45.5)	2.50± 1.14
Artisan	87	44 (50.6)	3.30±1.67	56 (64.4)	5.89±1.94	36 (41.4)	2.52±1.12
Farmer	5	4 (80.0)	3.20±1.62	2 (40.0)	8.04±0.83	2 (40.0)	3.19±1.99
Total	349	203 (58.2)	3.25±1.80	222 (63.6)	5.90±1.85	160 (45.8)	2.65±1.16

Figures in parentheses indicate percentages. SD=Standard deviation

Without a definite trend, maternal morbidity during pregnancy was found to be significantly more in women with lower educational level, except for *H. pylori* infection which was reported in significantly (p<0.05) higher proportions in women with higher educational level (Table 3). No definite trend of the impact of maternal education on foetal outcomes was observed, although most adverse outcomes were absent in women without formal education (Table 3). In the same vein, neither maternal morbidities nor foetal outcomes showed any specific trend in relation to maternal living accommodation, although women whose living accommodation was single rooms experienced more concomitant illnesses than women from other living accommodations (Table 3).

**Table 3.** Prevalence of adverse maternal and foetal outcomes in relation to maternal sociodemographic data*

Maternal sociodemographic data	Maternal outcomes					Foetal outcomes			
	Anaemia	Illnesses	*H. pylori*	HTN	DM	Assisted delivery	Surgical delivery	LBW	Post-term
Educational level									
None	87.5†	37.5†	25†	14.3†	-	-	-	12.5	-
Primary	71.4††	64.3††	16.7††	21.4††	2.5††	15.4^††^	37.7†	15.4	5.1
Secondary	59.9¶	58.5††	19.8††	7.7¶	1.8¶	6.9^¶^	5.0††	12.0	8.2
Tertiary	64.2¶	68.8††	33.3¶	13.7†	7.8§	10.2††	4.6††	17.6	6.5
Total	69.3	61.2	24.3	11.6	3.9	8.9	5.1	14.4	7.0
Living accommodation									
Single room	63.0	63.0†	20.1	12.0	3.8	9.9†	4.7†	12.9†	7.6
Flat	65.2	61.0†	29.6	11.1	3.8	8.7†	5.5†	12.6†	6.3
Bungalow	66.7	45.8††	20.8	8.7	4.3	4.8††	9.5††	33.3††	4.8
Total	64.1	61.0	23.9	11.4	3.8	9.1	5.3	14.2	6.9
Occupation									
Housewife	56.6†	64.2†	22.6†	3.8†	1.9†	5.9†	7.8†	18.0†	10.0
Civil servant	65.0††	69.7†	25.9†	16.3††	5.8††	8.6†	7.0†	18.8†	7.0
Artisan	60.9††	54.7††	24.1†	12.8††	3.5¶	8.4†	2.4††	6.0††	4.8
Student	68.9††	45.8¶	19.7†	6.9†	1.8†	15.1††	3.8††	11.3¶	7.5
Farmer	100.0¶	80.0§	40.0††	-	-	-	-	20.0†	-
Total	63.9	61.2	24.1	11.7	3.8	9.1	5.3	14.1	6.9

*Values are expressed as percentages. Values with different superscript are statistically different (p<0.05)

DM=Diabetes mellitus; HTN=Hypertension; LBW=Low birthweight

With the exception of pregnancy-induced hypertension found in significantly (p<0.05) higher proportions in women whose occupation was civil service, women whose occupation was farming had a significantly higher prevalence of anaemia, *H. pylori* infection, and other concomitant illnesses (Table 3). Significantly more adverse foetal outcomes were recorded in women who were housewives and farmers when compared with women who were civil servants and artisans.

## DISCUSSION

There seemed to be a wide nutritional disparity in this population as evidenced by wide variations in the levels of trace elements. Although the reason for this disparity is obscure, contamination of water source cannot be ruled out as contamination of water supply has been found to be one of the causes of acute toxicity of trace elements in the general population (23). Again, differential bioavailability of trace elements due to nutrient-nutrient interactions (24, 25) may be a contributory factor to the wide differences in concentrations of trace elements in this population. This has important public-health implications for mothers and their infants as this may reflect in differential plasma levels of these elements and maternal and foetal outcomes.

This study has documented a significantly higher prevalence of maternal morbidities (malaria, upper respiratory tract infection (UTI), anaemia, UTI plus malaria) among women from disadvantaged socioeconomic status as represented by women whose occupation was farming, women without formal education or primary education, and women whose living accommodation was single room, thus suggesting that maternal socioeconomic status impacts negatively on maternal health during pregnancy. This is consistent with the significant roles played by psychosocial factors, such as poverty and socioeconomic status in pregnancy outcomes earlier reported by Chandra (1).

In developing countries, socioeconomic status is a complex term generally used for defining social inequalities and usually measured by income/educational level/occupation/living accommodation. However, maternal hypertension was recorded in a higher proportion of civil servants/artisans. This suggests that civil servants and women who were artisans in this environment were more exposed to risk factors for hypertension (including stress) than other occupational groups. Stress has been related to hormonal changes, and occupational strain may result in shortened duration of pregnancy and babies who were small for their gestational age (26, 27).

The role of social factors on pregnancy outcomes, such as preterm, LBW, spontaneous abortion, alterations in foetal development, and long-term health of offspring, have been widely acknowledged (1). Significantly more post-term deliveries among housewives, more surgical deliveries among housewives and civil servants, and significantly more deliveries of LBW babies among civil servants and farmers respectively showed that housewives, farmers and civil servants were at increased risk of adverse foetal outcomes. While higher post-term delivery among housewives may be partly attributed to maternal inactivity, higher surgical delivery among housewives and civil servants may be due to maternal awareness and access to medical care (28). Delivery of LBW babies among farmers and civil servants may be partly attributed to either stress or/and nutritional deficiencies as evidenced by lower plasma levels of copper, iron, and zinc in women who were civil servants in the present study. It has been suggested that women who are employed as exemplified by civil servants/farmers may be at a risk of nutritional deficiencies because of reduced time for shopping and cooking (29). Moreover, working women who worked standing are less likely to eat three meals per day than women who worked in occupation that did not require long standing (30).

Deficit of trace elements, including zinc deficiency, has been associated with LBW (31). Additionally, increased physical activity, as measured by work in farms or fetching of water, for example, has been associated with LBW infants, smaller head-circumference, smaller mid-upper arm circumference, and lower placental weight (32). For example, maternal stress due to poverty is associated with preterm delivery via increased levels of epinephrine (33), increased oxytocin (34), and change in maternal habits (35).

Although maternal education did not display any trend in relation to maternal morbidities, the higher prevalence of maternal morbidities, such as anaemia, malaria, and UTI in women with lower educational level is in agreement with earlier studies (8). This is quite understandable as educational attainment has been established as a social variable that often displays the largest socioeconomic influence (36) because it affects both income and occupation. Educated women are also more likely to understand public-health message (37) and to maintain high personal hygiene than less-educated women. Educated women also belong to high social class and have access to adequate medicare and nutrition during pregnancy. However, a higher prevalence of *H. pylori* infection among women with higher educational level in the present finding supports the urban nature of the organism (38).

Ironical as it may sound, women without formal education had the lowest adverse foetal outcomes when compared with women with one form of education or the other. This is in contrast to other findings (2, 5) where women with the lowest education level had almost twice the odds of delivering a small-for-gestational age newborn compared to mothers in the highest education-category. The reason for this disparity is not clear but it may not be unconnected with the number of subjects (n=8) when compared with other groups. Koupilova *et al.* reported a birthweight gradient between women with primary and tertiary education (38).

A higher incidence of maternal morbidities and adverse foetal outcomes was reported in women whose living accommodation was a single room than other accommodation groups who also affirmed the role of maternal socioeconomic status on pregnancy outcomes. Although, in this population, living accommodation of women may not accurately indicate maternal socioeconomic status because of family dislocation due to job locations of spouses, women in lower socioeconomic strata have more adverse pregnancy outcomes (1).

In the present study, although maternal socioeconomic status appeared to influence maternal morbidity and pregnancy outcomes (via trace element nutriture), the effects were not clearly defined. This may be due to indices of maternal socioeconomic status used. For example, most women who were living in a single room were from higher educational level, and the reason given for such living accommodation was family dislocation as a result of job locations between couples. A high rate of unemployment may also contribute to invalidating maternal educational status—an indicator of socioeconomic status in the present study as some women educated to tertiary level still remained unemployed and lived in a single room. We, however, suggest that, in socioeconomically-disadvantaged population of developing countries, maternal occupation may be the more appropriate indicator for the assessment of maternal socioeconomic status.

## ACKNOWLEDGEMENTS

The authors acknowledge the staff of the Department of Obstetrics and Department of Gynaecology and Medical Records of the Federal Medical Centre for logistic support.
